# Paradoxical Herniation After Unilateral Decompressive Craniectomy Predicts Better Patient Survival: A Retrospective Analysis of 429 Cases

**DOI:** 10.1097/MD.0000000000002837

**Published:** 2016-03-07

**Authors:** Weiqiang Chen, Jingfang Guo, Jin Wu, Guoyi Peng, Mindong Huang, Chuwei Cai, Yingming Yang, Shousen Wang

**Affiliations:** From the Department of Neurosurgery, Fuzhou General Hospital of Nanjing Command, PLA, Fuzhou (WC, SW); Department of Neurosurgery, First Affiliated Hospital, Shantou University Medical College, Shantou (WC, JG, JW, GP, YY); Department of Neurosurgery, Jieyang People's Hospital, Jieyang (MH); and Department of Neurosurgery (CC), Shantou Central Hospital, Shantou, Guangdong, China.

## Abstract

Paradoxical herniation (PH) is a life-threatening emergency after decompressive craniectomy. In the current study, we examined patient survival in patients who developed PH after decompressive craniectomy versus those who did not. Risk factors for, and management of, PH were also analyzed.

This retrospective analysis included 429 consecutive patients receiving decompressive craniectomy during a period from January 2007 to December 2012. Mortality rate and Glasgow Outcome Scale (GOS) were compared between those who developed PH (n = 13) versus those who did not (n = 416). A stepwise multivariate logistic regression analysis was carried out to examine the risk factors for PH.

The overall mortality in the entire sample was 22.8%, with a median follow-up of 6 months. Oddly enough, all 13 patients who developed PH survived beyond 6 months. Glasgow Coma Scale did not differ between the 2 groups upon admission, but GOS was significantly higher in subjects who developed PH. Both the disease type and coma degree were comparable between the 13 PH patients and the remaining 416 patients. In all PH episodes, patients responded to emergency treatments that included intravenous hydration, cerebral spinal fluid drainage discontinuation, and Trendelenburg position. A regression analysis indicated the following independent risk factors for PH: external ventriculostomy, lumbar puncture, and continuous external lumbar drainage.

The rate of PH is approximately 3% after decompressive craniectomy. The most intriguing findings of the current study were the 0% mortality in those who developed PH versus 23.6% mortality in those who did not develop PH and significant difference of GOS score at 6-month follow-up between the 2 groups, suggesting that PH after decompressive craniectomy should be managed aggressively. The risk factors for PH include external ventriculostomy, ventriculoperitoneal shunt, lumbar puncture, and continuous external lumbar drainage.

## INTRODUCTION

Decompressive craniectomy (DC) is a life-saving emergency surgery in patients with intracranial hypertension uncontrollable with other means.^1–3^ However, the conversion of the cranial case from a “closed” to an “open” box carries a variety of risks. Among these complications, paradoxical herniation (PH; herniation of intracranial contents infratentorially and compression of brainstem at the foramen magnum along with brain gravity) is rare, but potentially fatal.^3–6^ Due to its paucity, PH has only been reported as sporadic case reports and case series.^3–6^ In the current study, we retrospectively reviewed 429 consecutive cases of DC, and compared patient survival in those who developed PH versus those who did not. We also attempted a multivariate logistic regression analysis to determine the risk factors for PH. Basic clinical features and management were also reviewed.

## MATERIALS AND METHODS

### Study Design

The current study was approved by the Ethics Committee of all 4 participating hospitals (Fuzhou General Hospital, First Affiliated Hospital of Shantou University Medical College, Jieyang People's Hospital, and Shantou Central Hospital). Written consent was given by the patients or their family for their information to be stored in the hospital database and used for research purposes. The current study included all 429 patients receiving unilateral DC at the 4 participating hospitals during a period from January 2007 to December 2012.

Briefly, all the patients receiving unilateral DC met the following criteria as previously described^7–10^: coma with Glasgow Coma Scale (GCS) of 8 or less at admission, and midline shift >5 mm and compressed basal cisterns on computerized tomography (CT) scans, sustained intracranial hypertension at 20 to 35 mm Hg despite medical management.

Cases with the following conditions were excluded from data analysis^7^: multiple injury, previous disabling neurological disease, previous craniectomy, spinal cord injury, penetrating brain injury, fixed dilated pupils, and GCS score of 3.

### Management Procedures

DC was carried out in the frontoparietotemporal region, based on the lesion location and midline shift determined by CT scans.^11,12^ The boundaries of DC were: anterior—midpupillary lines; posterior—3–4 cm posterior to the external acustic meatus; superior—2 cm of the lateral edge of the superior sagittal sinus; inferior—the level of the zygomatic arch.

Postoperative treatments were similar to that previously described,^7,13–16^ and included elevation of the upper part of the body by 15° to 30°, assisted ventilation to ensure oxygen saturation of 95% or more, and arterial carbon dioxide at about 40 mm Hg, sedation and muscle relaxation if necessary, and control of blood glucose and body temperature.

The minimal follow-up was 6 months. To examine the risk factors for PH, a stepwise multivariate regression analysis was carried out based on the results of preliminary univariate regression, as previously described.^17^ The diagnosis of PH was based on clinical findings and imaging results showing mechanical displacement of the brain away from the side of craniectomy. Those with mechanical displacement of the brain towards the side of craniectomy, with no mechanical displacement, were excluded from PH.

### Statistical Analysis

Continuous variables (except age) are presented as median and interquartile range (IQR), and analyzed using Mann–Whitney *U* test. Categorical data (eg, mortality) are presented as percentage, and analyzed with chi-square analysis or Fisher exact test. Statistical significance was set at *P* < 0.05. All statistical analyses were performed using SPSS 17.0 (SAS Institute Inc., Cary, NC).

## RESULTS

### General Information

The cohort consisted of 303 men and 126 women. The mean age was 57.9 years (range 4 months to 75 years). The causes for DC in patients who did not develop paradoxical herniation included the following: hemorrhagic stroke (n = 242), trauma (n = 172), and malignant glioma (n = 2). Table 1  shows the main characteristics of the 13 patients with PH (for a total of 14 episodes). The underlying conditions of the 13 patients with PH were generally consistent with the overall sample (*P* = 0.81) (n = 5 for trauma, n = 8 for hemorrhagic stroke). Disease severity, as reflected by GCS on admission, did not differ between those who developed PH versus those who did not (6.38 ± 1.26 vs 6.93 ± 3.42; *P* = 0.18).

**TABLE 1:**
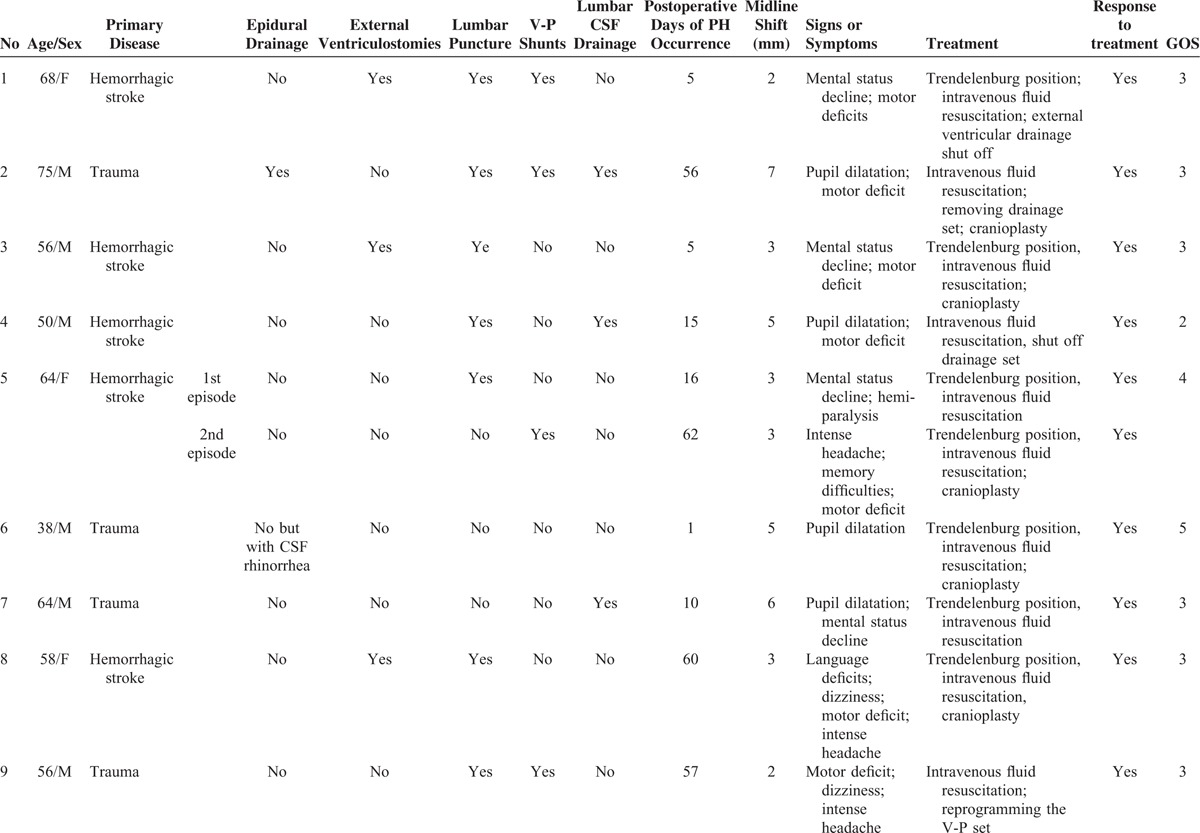
The Characteristics of 13 Patients With Paradoxical Herniation

**TABLE 1 (Continued):**
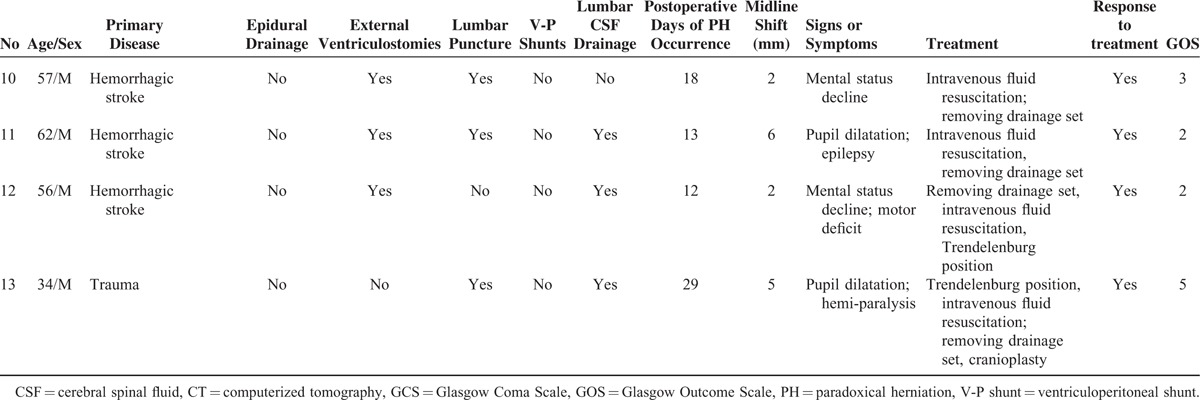
The Characteristics of 13 Patients With Paradoxical Herniation

**FIGURE 1:**
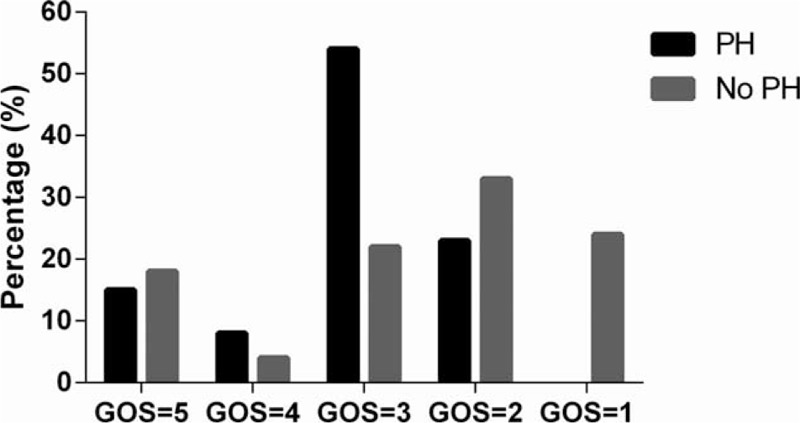
Glasgow Outcome Scale (GOS) at 6-month follow-up (% for each category). Mann–Whitney *U* test: significant difference between the PH group and the no PH group (*P* = 0.048); GOS was defined as follows: 1 = death, 2 = persistent vegetative state, 3 = severe disability, 4 = moderate disability, and 5 = good recovery. PH = paradoxical herniation.

The mortality rate in the overall sample was 22.8%. The diameter of bone window did not differ between those who developed PH versus those who did not (10.94 ± 0.92 vs 11.25 ± 1.09 cm; *P* = 0.242). All 13 patients who developed PH survived to be discharged (and for at least 6 months). A chi-square test showed a reduced mortality (*P* = 0.046) and a better clinical outcome (*P* = 0.048) in those who developed PH (Figure 1).

**FIGURE 2:**
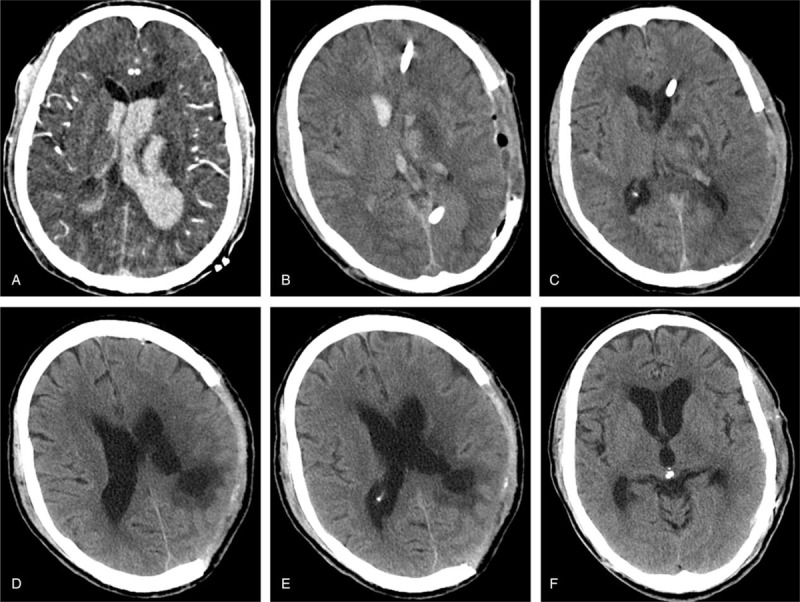
Imaging features of a representative case of paradoxical herniation. A, CT and Computed tomography angiography (CTA) upon admission, showing left occipital hematoma rupturing into the ventricular system and no vascular malformation. The GCS score was 9. A hematoma in the occipital lobe was removed. The decompressive craniectomy (left side) was followed by ventriculostomy. ICP returned to normal range after the surgery. B, CT scan postoperative day (POD) 1. C, CT scan on POD 5. At this time, the neurologic status deteriorated. ICP was 1 to 3 mm Hg. Systolic BP decreased to 75 mm Hg. External ventricular drainage was shut off, and the patient was placed in the Trendelenburg position with rapid intravenous hydration. D, CT scan on POD 14. CT = computerized tomography, GCS = Glasgow Coma Scale, ICP = intracranial pressure.

**FIGURE 3:**
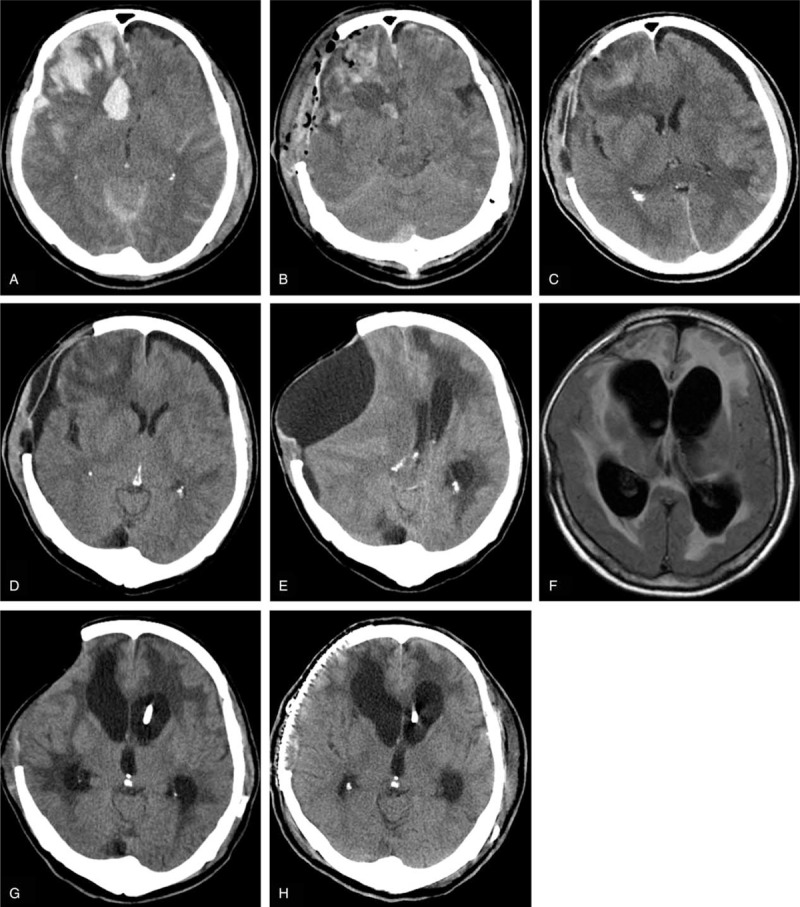
A representative case with paradoxical herniation at 2 weeks to 2 months, showing sinking skin flap. A, CT scan revealing a hemorrhagic contusion in the right frontotemporoparietal region and diffuse brain swelling upon admission. B, CT scan immediately after emergency decompressive craniectomy. Lumbar puncture was performed daily to remove 20 mL CSF per day. C, CT scan on postoperative day (POD) 10, indicating a subdural hygroma in the left frontal region. The patient did not respond to dehydration and lumbar puncture. D, CT scan on POD 30. The patient developed an enlarged subdural hygroma in the left frontal region and a subgaleal fluid collection in the right frontal region after persistent CSF lumbar drainage. E, CT scan on POD 56, showing increased subgaleal fluid accumulation with flattening of overlying cortex and significant midline shift. The GCS was 4. A diagnosis of paradoxical herniation was established, and the external lumbar drainage was weaned. He was placed in the Trendelenburg position and received rapid intravenous hydration. F, MRI on POD 106, showing markedly reduced subdural hygroma and resolution of midline shift. G, Ventricular-peritoneal shunt for hydrocephalus (on POD 122). H, cranioplasty on POD 137. CSF = cerebral spinal fluid, CT = computerized tomography, GCS = Glasgow Coma Scale, ICP = intracranial pressure, MRI = magnetic resonance imaging.

In 8 out of the total 14 episodes, PH occurred between 2 weeks and 2 months after the craniectomy; in the remaining 6 episodes, PH occurred within 2 weeks after craniectomy. A notable difference between the cases within versus after 2 weeks was tense, but not sinking skin flap at the site of DC within 2 weeks, versus sinking skin flap after 2 weeks. Other symptoms/signs included the following: motor deficit (n = 8), pupil dilatation (n = 6), intense headache (n = 3), hemiparalysis (n = 2), and epilepsy (n = 1) (Figures 2 and 3).

### Imaging Features and Intracranial Pressure

Computerized tomography revealed midline shift away from the side of craniectomy and brainstem compression in all PH episodes. The following structural alterations suggesting intracranial hypotension were also noted: flattening of the pons along with shrinking or effacement of prepontine and perichiasmatic cisterns in 8 cases and subdural hygromas in 7 cases.

The blood pressure during the episodes of PH was as follows: 72 to 81/35 to 46 mm Hg. Intracranial pressure (ICP) was monitored in 4 patients using the Codman monitor for an average of 10 days. Low ICP was evident (ICP range: 1–3 mm Hg throughout the episodes). In 11 patients who underwent lumbar puncture, ICPs ranged from 2 to 5 cm H_2_O. In the 137 patients who received Codman monitoring, but did not develop PH, the ICP ranged from 12 to 79 mm Hg.

### Management of PH

Dehydrating agents (eg, mannitol) and cerebral spinal fluid (CSF) drainage were suspended in all 14 episodes. Intravenous hydration (2500–4000 mL/day) was implemented. Trendelenburg position was adopted in 7 patients. In the 2 patients with persistent CSF lumbar drainage, 20 to 50 mL saline was injected via the drainage tube slowly. In 1 of 3 patients with ventriculoperitoneal shunt, the shunt was reprogrammed from 160 mm H_2_O to 200 mm H_2_O valve setting. In all 14 episodes, the patients responded to the treatment and survived beyond the dissipation of PH.

**FIGURE 4:**
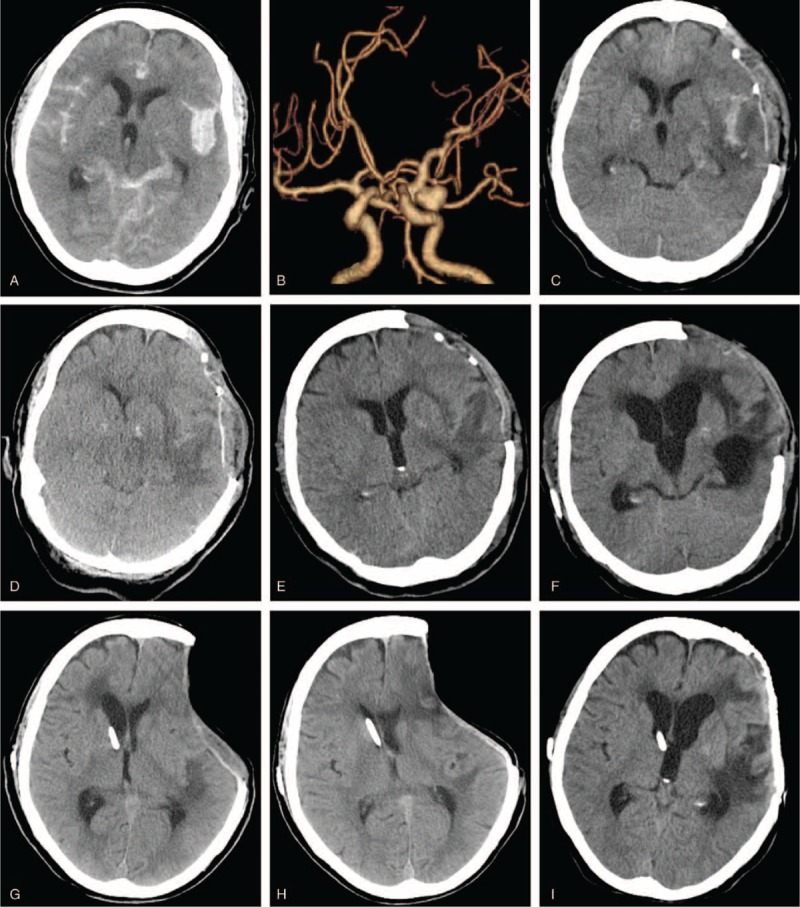
Imaging findings in the case with 2 episode of paradoxical herniation. A, CT scan showing subarachnoid hemorrhage (SAH), a hematoma in the left sylvian fissure, brain swelling, and no midline shift upon admission. B, CTA scan revealing an aneurysm at the terminal segment of the carotid artery. The patient underwent emergency decompressive craniectomy, followed by epidural drain. ICP returned to normal range. The patient developed high fever. On postoperative day (POD) 6, a lumbar puncture was performed, and provided evidence to rule out pyogenic meningitis. D, CT scan on POD 16, showing paradoxical herniation. At this time, the GCS score was 5; BP was 81/38 mm Hg; ICP was 2 cm H_2_O. The patient responded to rapid hydration and Trendelenburg position. E, CT scan on POD 23, showing resolution of the midline shift. On POD 36, neurological function deteriorated. F, CT scan showing enlarged ventricular system with external brain tamponade. The patient received emergency ventriculoperitoneal shunt. G, CT scan on POD 44 showing much smaller ventricular system. H, CT scan on POD 62, showing the second episode of paradoxical herniation. I, Cranioplasty on POD 65. The patient responded to treatment, and recovered to discharge. CT = computerized tomography, GCS = Glasgow Coma Scale, ICP = intracranial pressure.

At approximately 3 months after craniotomy, cranioplasty was carried out in 6 cases; the remaining 7 patients did not choose cranioplasty for personal reasons. The case with repeated PH after DC is shown in Figure 4. The first episode was managed successfully with rapid hydration and best rest in the Trendelenburg position. A second episode of PH occurred shortly after a ventriculoperitoneal shunt, and responded to treatments. The patients then received cranioplasty and recovered smoothly.

### Risk Factors

In the first step of the analysis (univariate regression), we examined the association between PH with demographical variables and a number of potentially relevant factors (Table 2). The results indicated an association with: external ventriculostomy (6/13 vs 13/416 in subjects with vs without paradoxical herniation), ventriculoperitoneal shunt (3/13 vs 19/416), lumbar puncture (11/13 vs 84/416), and continuous external lumbar drainage (6/13 vs 7/416). The second step of multivariate analysis confirmed the following 3 risk factors: external ventriculostomy, lumbar puncture, and continuous external lumbar drainage, with the following regression equation: 
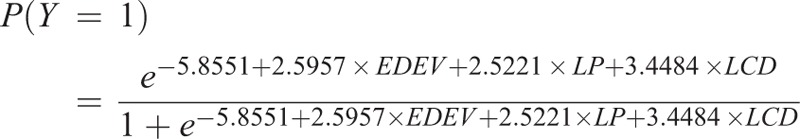


**TABLE 2:**
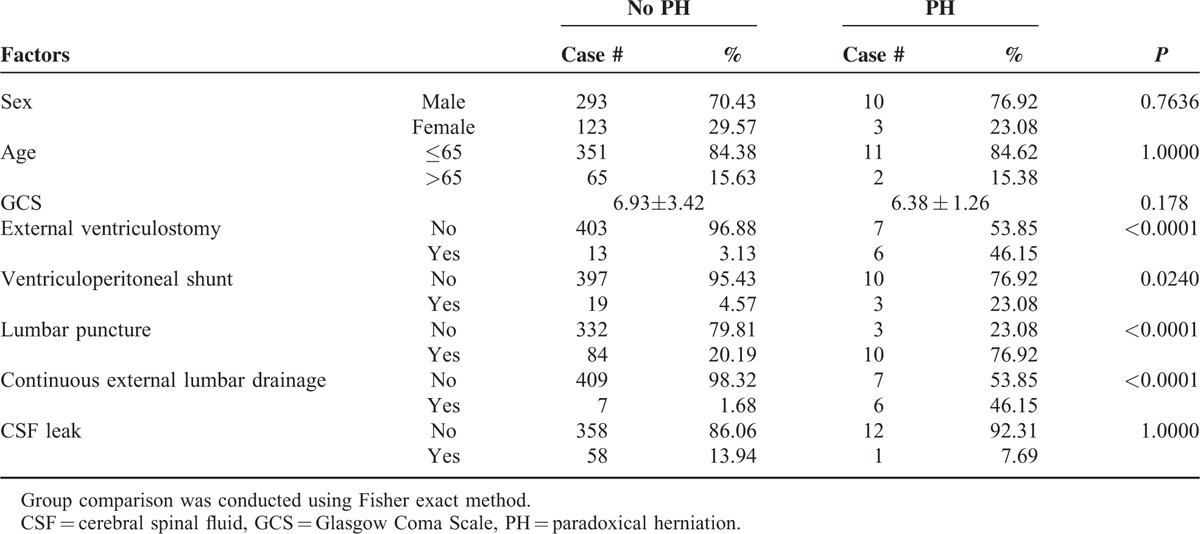
Univariate Regression Analysis

## DISCUSSION

Upon DC, the Monro–Kellie model is disrupted.^3^ This, in combination with altered CSF circulation,^18,19^ and cerebral blood flow,^20,21^ could reduce ICP and result in a complex symptom known as the syndrome of the trephined. Grant and Norcross^22,23^ first described the syndrome of the trephined in 1939. Since sinking skin flaps at the site of craniectomy is observed in majority of the patients, this syndrome is also known as the “sinking skin flap syndrome.”^24^ If untreated, this condition could lead to PH.^4^ In the current study, the rate of paradoxical herniation after DC is 3.0%. However, we observed sinking skin flap only in the 8 cases of paradoxical herniation that occurred beyond 2 weeks after craniectomy and the 6 cases that occurred within 2 weeks after craniectomy. This finding suggests that sinking skin flap at the site of craniectomy is neither necessary nor sensitive marker for paradoxical herniation. The absence of conspicuous sinking skin flap at 2 weeks after craniectomy likely reflects the subsiding brain edema.^3^

The most intriguing findings in the current study are as follows: 0 mortality in the subjects who developed PH versus 23.6% mortality in those who did not develop PH, and also better overall neurological outcomes at 6-month follow-up, despite of comparable disease type and severity upon admission. DC has been shown to decrease ICP.^25,26^ Polin et al^25^ reported a decrease of 66.4% in ICP after DC. Another trial involving 27 children by Simma et al^26^ in 2002 showed 84% decrease of ICP after DC. In the current study, we found very low ICP (ICP range: 1 to 3 mm Hg throughout the episodes) in the patients with PH versus 12 to 79 mm Hg in the 137 patients who received Codman monitoring, but did not develop PH. In the 34 patients with continual ICP monitoring who eventually died, the ICP fluctuated from 35 to 79 mm Hg. A limitation of the current study was lack of continual ICP monitoring in majority of the subjects, and as a result, we did not include ICP in the regression analysis. Consistent with previous study,^27^ low blood pressure (72–81/35–46 mm Hg) and high heart rate (105–135 bpm) in patients with PH, and imaging findings in the current study also supported intracranial hypotension.

Cerebral edema and particularly delayed edema lead to secondary injury to the brain after insults.^27,28^ There is a strong, and even linear association between increased ICP and poor outcome in patients with diffuse traumatic brain injury.^28,29^ Therefore, we believe that the 0% mortality in the subjects who developed PH (relative to 23.6% mortality in those who did not develop PH) reflects the low ICP, which in turn may reflect the lower severity of the lesions (at the time of PH occurrence rather than upon admission) and/or more successful management.

The current study included a stepwise logistic regression in an attempt to determine the risk factors for paradoxical herniation. In the first step of univariate analysis, paradoxical herniation was associated with external ventriculostomies, ventriculoperitoneal shunts, lumbar puncture, and continuous external lumbar drainage of CSF (Table 2), but not background diseases, sex, and age. A multivariate regression revealed the following independent risk factors: external ventriculostomies, ventriculoperitoneal shunts, lumbar puncture, and continuous external lumbar drainage of CSF, highlighting the critical role of CSF loss (and the resulting intracranial hypotension) in the development of paradoxical herniation, as suggested by previous studies.^3–6,19^ Similar to previous reports of paradoxical herniation secondary to prolonged CSF leakage through occult dural defects or intraoperative CSF drainage,^19,30^ 1 out of the 13 patients with paradoxical herniation in our series had CSF leak. The compression of subarachnoid space through the site of craniectomy by the atmospheric pressure could shift the entire brain (as suggested by the imaging findings in the current study) and interfere with the circulation of CSF, and eventually contribute to paradoxical herniation.^3^

Lumbar puncture is routinely carried out if meningitis is suspected. Conventional wisdom, and also the results of our multivariate analysis suggest that lumbar puncture may induce paradoxical herniation. Such a finding indicates the need to balance the need to verify the potential presence of meningitis and the risk of paradoxical herniation. The current study does not provide a practical guide on this issue, but we believe that the risk must be considered before lumbar puncture in patients with DC. A sinking skin flap is clearly a sign that requires close attention, but is not always present upon paradoxical herniation, and particularly beyond 2 weeks after craniectomy. The reasons for the lack of sinking skin flap are not known, but a previous study^31^ indicated that subdural/epidural hygroma could result in deceiving fullness to the overlying skin flap.

**FIGURE 5:**
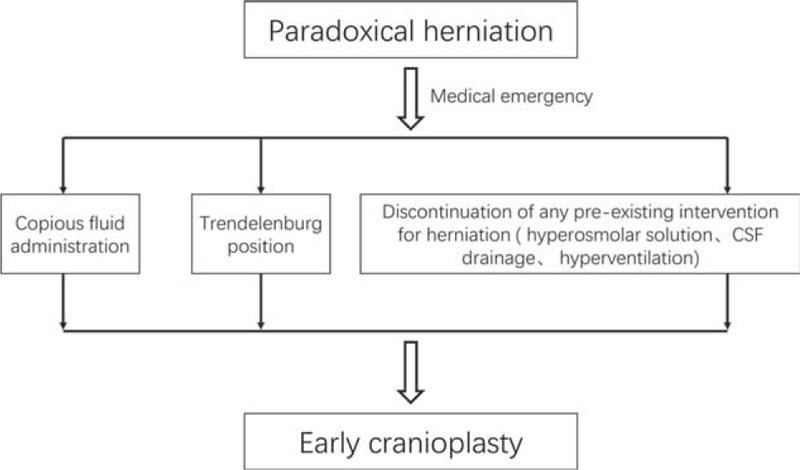
A flow chart of recommended protocol for treating paradoxical herniation (PH).

Early cranioplasty—conversion of an “open box” to “closed box”—is the ultimate solution (Figure 5), but is not possible in all cases. Cranioplasty improves cerebral blood flow in the defect area and prevents thinning of the cortical mantle.^32^ At a regional level, cranioplasty increases superior sagittal sinus pressure, CSF motion, cerebral metabolism, and cerebral vascular reserve capacities.^33^ The case with 2 episodes of paradoxical herniation in our series supports this notion. The first episode was managed with intravenous fluids and best rest in the Trendelenburg position. A second episode of paradoxical herniation occurred shortly after a ventriculoperitoneal shunt. This patient then received cranioplasty: no more episodes occurred afterwards.

## CONCLUSIONS

Paradoxical herniation occurs after DC at an approximate rate of 3%, most commonly between 2 weeks and 2 months after the surgery. The development of PH predicts better patient survival. As a result, we advocate aggressive management upon PH. Risk factors for paradoxical herniation include external ventriculostomy, ventriculoperitoneal shunt, lumbar puncture, and continuous external lumbar drainage of CSF.
